# Effects of Self-Care for Older PErsons (SCOPE) on Functional and Physiological Measures: A Cluster Randomized Controlled Trial

**DOI:** 10.3390/jcm9030885

**Published:** 2020-03-24

**Authors:** Ted Kheng Siang Ng, David Bruce Matchar, Rehena Sultana, Angelique Chan

**Affiliations:** 1 Center for Aging, Research and Education, Duke-National University of Singapore Medical School, Singapore 169857, Singapore; ted.ng@duke-nus.edu.sg; 2 Program in Health Services and Systems Research, Duke-National University of Singapore Medical School, Singapore 169857, Singapore; david.matchar@duke-nus.edu.sg; 3 Department of Medicine (General Internal Medicine), Duke University School of Medicine, Durham, NC 27710, USA; 4 Center for Quantitative Medicine, Duke-National University of Singapore Medical School, Singapore 169857, Singapore; rehena.sultana@duke-nus.edu.sg; 5 Department of Sociology, Faculty of Arts and Social Sciences, National University of Singapore, Singapore 117570, Singapore

**Keywords:** self-care, randomized control trial, community-based program, physiological measures, functional measures

## Abstract

Background: Population aging poses unprecedented demands on the healthcare system. There is also a scarcity of evidence on self-care intervention to improve objective measures of morbidity and aging-associated functional and physiological measures in a low-income multi-ethnic population setting. Methods: We conducted a cluster randomized controlled trial (ClinicalTrials.gov Identifier: NCT01672177) to examine the effects of the Self-Care for Older PErsons (SCOPE) program. We randomized 14 Senior Activity Centers and randomly selected older adults within these centers. Functional and physiological measurements were performed at baseline, 10-month, and 18-month periods. The primary outcome was a composite of three morbidity-specific measures, which include hemoglobin A1c (HbA1C), peak expiratory flow, and systolic blood pressure. Aging-associated functional and physiological measures were examined as secondary outcomes. Repeated-measure mixed models were employed to examine the effects of SCOPE on these measures. Results: 378 community-dwelling older adults participated in either the treatment (*n*= 164) or the control arm (*n* = 214). The primary outcome was not significantly improved. For the secondary outcomes, SCOPE participants demonstrated slower oxygen desaturation at an 18-month period (*p* = 0.001), improved time to complete the chair-stand test (*p* < 0.001) at a 10-month period with the effect persisting at the 18-month period (*p* < 0.001). SCOPE participants also had significantly improved vitamin B_12_ levels at the 18-month period (*p* < 0.001), increased hemoglobin concentration (*p* < 0.001), decreased mean corpuscular volume (*p* = 0.001), and decreased creatinine (*p* = 0.002) at the 10-month period. Conclusions: SCOPE did not improve morbidity-specific measures. However, it improved several aging-associated measures implicated in geriatric syndromes. This study highlights the potential of a self-care program in the prevention of geriatric syndromes in community-dwelling older adults, while emphasizing self-management to manage existing morbidities.

## 1. Introduction

Currently, there is a shortage of geriatricians and developing community care services at an accelerated pace to enable aging in place that is in dire need. A major driver of the success of community care services is empowering older adults to acquire self-care skills to manage chronic conditions. Chronic Disease Self-Management Programs (CDSMPs) in other settings have shown encouraging findings. Self-rated health improved, and healthcare utilization decreased significantly with fewer hospitalizations and fewer days spent in the hospital [1,2]. CDSMP programs targeted at improving biomarkers of morbidities have yielded equally promising findings. A program targeted at diabetic patients showed significantly reduced hemoglobin A1c (HbA1c) levels after intervention [3] and improved peak expiratory flow and blood pressure [4,5,6,7]. However, these CDSMP programs were conducted in hospital settings and participants were more likely to be motivated to manage their conditions.

Improving self-care could be a more sustainable model. The concept of self-care is based on the self-efficacy theory framework [8,9]. Self-care involves improving coping resources to help older adults be their agents of change in managing the aging process and to actively manage their health conditions [10]. Self-care aims to enhance general health knowledge, such as nutrition, exercise, and engagement with the healthcare system [11]. Different from self-management, which is the core of CDSMP, self-care is a multi-dimensional concept. Refer to the routine coping with the effects of aging, not solely relying on CDSMP. Although both are anchored in the self-efficacy model, self-management focuses on improving specific conditions in particular while self-care has a broader target and effects. Although CDSMP has been well-studied, less is known regarding the effect of self-care programs on chronic diseases and dysregulated aging physiology implicated in older adults, especially in deprived populations of low socioeconomic status and ethnic minorities. Another rationale to examine a self-care program is the difficulty present with the current delivery of CDSMPs in that patients have to be diagnosed and physically present in the hospital for the intervention. However, most community-dwelling older adults are undiagnosed for diabetes, hypertension, or chronic obstructive pulmonary disease (COPD) and, thus, unlikely to enroll in a CDSMP. Lastly, while self-care ability is an important health asset [12], inadequate self-care knowledge was shown to be related to socio-economic characteristics [13,14].

Given these gaps of knowledge, we evaluated a self-care program, named the Self-Care for Older PErsons (SCOPE) intervention—a broadly-applicable, empowering, and engaging self-care program, using a cluster randomized controlled trial (RCT) design in the communal setting. As we aimed to examine the effect of this broad-based interventional program on low-income older adults, which often have high heterogeneity in terms of morbidities, we included participants both with and without common morbidities. The primary outcome measure was a composite measure comprising three measures including morbidity indicators for hypertension, diabetes, and COPD. The primary hypothesis of this study was that SCOPE would elicit meaningful change on disease-specific composite measures of hypertension, diabetes, and COPD in multi-ethnic, low-income, and community-dwelling older adults in at least one of the morbidity markers of the three conditions (i.e., blood pressure, HbA1c, or peak flow). Furthermore, aiming to investigate the broad effects of self-care on geriatric syndromes, our secondary hypothesis was that SCOPE would improve objective functional and physiological measures that are dysregulated in aging.

## 2. Methods

### 2.1. Study Design

This study was approved by the (National University of Singapore) Institutional Review Board (National University of Singapore) -IRB Reference No: 11–111) and registered at the ClinicalTrials.gov (https://clinicaltrials.gov/ct2/show/NCT01672177?term=scope+self-care&cntry=SG&draw=2&rank=2). This study is a parallel-arm cluster RCT (ClinicalTrials.gov Identifier: NCT01672177) with the intervention sites as the unit of randomization. The intervention sites were 14 Senior Activity Centers (SACs) selected from a total of 42 in Singapore. The SACs were paired with matching based on similarities in age, ethnicity, and proportion of gender served by the SAC. One SAC in each pair was assigned as either the intervention or the control arm based on a coin-flip.

### 2.2. Subject Recruitment

We obtained a list of individuals attending each SAC and randomly selected potential participants using a computer random number generator. Informed consent was obtained before the screening process of eligible participants took place. We administered screening interviews in English, Malay, and Chinese. Inclusion criteria included being aged 55 or above, affiliation with one of the SACs, absence of cognitive impairment based on the Abbreviated Mental Test (AMT), no debilitating or terminal illness, and no activities of daily living (ADL) disability that rendered the subject unable to participate in the intervention. Exclusion criteria included an unwillingness to have a blood draw, currently on chemotherapy or dialysis, or receiving medications for major psychiatric illness.

### 2.3. SCOPE Intervention

We applied two conceptual frameworks in guiding the aims and content of the intervention. First, we adopted concepts from Bandura’s self-efficacy framework [15,16,17] to influence older adults’ beliefs in their own capacities to exercise control over their own functioning. Second, the intervention was based on empowerment theory [18], which advocates the development of older adults’ skills and resources for gaining control over their lives. More specifically, the content of the intervention materials focused on health promotion, disease prevention, health seeking behavior, chronic disease management, and anti-stigmatization. The session topics included: successful aging, basic anatomy, personal health goals, nutrition and exercise, chronic diseases management including medication and prevention, access to healthcare facilities, self-efficacy, stress management, and psychological well-being and alternative medicine.

From 2011–2014, the intervention sessions were held in person at the respective SACs weekly over 10 months, with each session spanning approximately two hours. From 10 to 18 months, no intervention was conducted. One community health worker (CHW) was assigned to each SAC intervention site. The intervention was provided by CHWs who had previously been trained by trainers from the Tsao Foundation. The Tsao Foundation trainers were community nurses who had many years of experience working with older adults to develop skills for mastering the aging journey. The CHWs provided the participants with monthly health journals with action items and pictures to take home and review. Three versions of the health journals were available in English, Malaysian, and Chinese. Depending on the language preference of each SAC, the intervention was delivered in one of the three major languages spoken in Singapore, including English, Malaysian, and Mandarin.

### 2.4. Control Group

Participants in the control arm did not receive any of the materials provided in the intervention sessions. However, they were required to fill in their health journals each month, complete questionnaires, and participate in three health screenings and blood draw at baseline, 10-month, and 18-month periods.

For both intervention and control groups, the interviewers were blinded to the participants’ study arm. Data on psychosocial and functional measures and a blood draw were collected at baseline, 10-month, and 18-month periods. The detailed protocol of this trial was published previously [19].

## 3. Outcome Measures

### 3.1. Primary Outcome Measure

The primary outcome measure was based on a composite of indicators for three conditions: systolic blood pressure (hypertension), HbA1C (diabetes), and peak expiratory flow (COPD). Hypertension was deemed present if the average blood pressure over three measurements exceeded 140/90 mmHg for participants without diabetes, or 130/80 mmHg for those with diabetes. HbA1c over 6.5% was used as an indicator of diabetes [20,21]. Respiratory obstruction, which is a characteristic of COPD, was defined as PEF of less than 80% of the expected value on the highest of three trials [22,23]. Expected values were calculated using a standard formula and adjusting for age, gender, and height.

### 3.2. Secondary Outcome Measures

#### 3.2.1. Functional Measurements

We assessed seven aging-implicated functional measures. The participants performed the chair-stand test by standing up straight from the chair as quickly as they can, without stopping in between, and keeping their arms folded across their chests. The measure for the chair-stand test was the time for a total of five stands, recorded to the hundredths of a second. Hand-grip strength was measured by using a Smedley spring-type dynamometer (Hand Grip Meter, No. 6103, TANITA, city, Tokyo, Japan, 75 kg). The participants were instructed to squeeze the handle as hard as they could for a couple of seconds and then let go. Questionnaires on independent-activities of daily living (ADL) and self-rated health were administered as well.

#### 3.2.2. Physiological Measurements

A total of 18 physiological measures were assessed, including body-mass index (BMI), waist circumference, pulse, blood pressure, HbA1C, Vitamin B_12_, hemoglobin, mean corpuscular volume (MCV), platelets, white blood cell (WBC) count, total cholesterol, high-density lipoprotein (HDL) cholesterol, low-density lipoprotein (LDL) cholesterol, triglycerides, albumin, creatinine, urine micro albumin, and urine creatinine.

## 4. Statistical Analyses

### 4.1. Sample Size Calculation

We based our sample size calculation on the anticipated prevalence of diabetes (35%), hypertension (70%), and chronic obstructive lung disorder (COPD) [24,25] in the study population. The COPD difference in means was not included in the sample size calculation due to the lack of information regarding the expected change in peak flow readings. The intra-cluster correlation used was 0.01 for systolic blood pressure and 0.04 for HbA1c. Power of the study was set at 80% and an alpha level at 5%. Accounting for an anticipated attrition rate of 20%, the required sample size was estimated to be 378 participants.

The equation used for the sample size calculation [26] is shown below.

(1) $$m=\left(1-\rho \right)/\left(\frac{k_{max}}{k_{\left(m=1\right)}}-\rho \right)$$

where,

(2) $$k_{\left(m=1\right)}=\frac{{\left(t_{\frac{\alpha }{2}}+t_{\beta }\right)}^{2}\left(2\sigma^{2}\right)}{{\left(\mu_{1}-\mu_{2}\right)}^{2}},\;df=2\left(k_{max}-1\right)$$

*m* = number of participants in each cluster
*k_max_* = the maximum number of clusters that can be assigned in each arm
*k*_(*m*=1)_ = the number of clusters in each arm that would be required if *m* = 1
*µ*_1_ = population mean in treatment arm 1
*µ*_2_ = population mean in treatment arm 2
*µ*_1_ − µ_2_ = the difference in means between the two arms to detect
*σ*^2^ = population variance
*ρ* = intra cluster correlation coefficient
*t*_α/2_ = critical value for *α* = 0.05
*t*_β_ = critical value for *β* = 0.2

### 4.2. Primary Composite Measure

At each time-point, each indicator was coded as 1 if it fulfilled the diagnostic criteria. Otherwise, it was coded as 0. Then, the composite measure for each participant at each time-point was calculated based on the sum of the three coded indicators. Subsequently, the score differences between two immediate time-points were computed, with improvement in ≥1 measure(s) over the two time-points coded as 1 and no improvement or worse condition(s) coded as 0. The composite measure was designed to identify meaningful changes in any one of the three indicator conditions, and was calculated via simulation to account for participants who either did not have any one of the target conditions or who had them controlled.

### 4.3. Secondary Measures

We used Bonferroni’s correction to account for multiple comparisons. It has been the most widely used approach for controlling a type I error rate that could arise from multiple tests, especially when multiple hypotheses are being tested simultaneously and are highly correlated [27,28,29].

As per nature of the data, the primary outcome measures were expressed as the number of cases and percentage, while the secondary measures were expressed as mean ± standard deviation (SD). The differences in baseline variables were examined using Student’s *t*-test, chi-square, or Fisher’s exact tests. A repeated-measure mixed model was employed to examine treatment effects on the outcomes by comparing the difference-in-difference between the two groups. This model takes into account missing data without imputation or mean substitution when there were missing data at one or more time-points for a particular participant. This statistical model, thus, has the advantage of producing robust results for longitudinal data, which inevitably incur loss-to-follow-ups [30]. Baseline values of the respective outcome variables, age, gender, ethnicity, time-points of the intervention, treatment arm, and interaction term of time-points and treatment arm were included as covariates in the model as covariates. Analyses were performed using the Statistical Package for the Social Sciences (SPSS) version 24.0 (IBM SPSS Statistics for Windows, Version 24.0., Armonk, NY, USA) with two-tailed *α*-value of <0.05 considered as statistically significant.

## 5. Results

### 5.1. Baseline Demographics and Characteristics

We recruited a total of 378 participants between the ages of 55–95 years (mean = 72.19 years, SD = 8.03). Treatment and control arms’ allocation ratio was not 1:1. This imbalance was due to the different numbers of recruited participants in each matched study site. The participants were matched based on age, gender, and ethnicity. No significant differences in baseline and demographic variables were observed, except ethnicity (more Chinese in the intervention group, *p* = 0.032) and marital status (fewer married individuals in the intervention group, *p* < 0.001) (Table 1). Thus, ethnicity and marital status were included in subsequent modeling to account for selection bias. At baseline, *N* = 157 (97.5%) in the intervention group and *N* = 203 (96.7%) of the participants in the control group had at least one of the three SCOPE-targeted conditions (Table S1). Approximately 75% of the participants completed all the intervention sessions.

### 5.2. Effects of SCOPE on Primary Outcome

There was no difference between the intervention and control subjects on the composite measure of improvement in at least one of the morbidity indicators (*p* = 0.795 for 10-month and *p* = 0.459 for 18-month period). Sensitivity analyses were also performed and yielded similar results (Table 2). Additionally, there was also no evidence at the study arm level of differences in mean HbA1C levels, blood pressure (systolic or diastolic), or peak expiratory flow across the time-points (Table S2).

### 5.3. Effects of SCOPE on Secondary Outcomes: Functional and Physiological Measures

Since we needed to perform 11 fully adjusted repeated-measure linear-mixed models based on the preliminary results of the unadjusted models (Table S2), Bonferroni’s correction was applied, yielding a threshold of <0.005 defining statistical significance for the subsequent fully adjusted models. After accounting for multiple comparisons using Bonferroni’s correction, we observed statistically significant differences in eight measures (Table 3 and Table 4). These significant measures included the total time to complete the chair-stand test and oxygen desaturation upon completing the chair-stand test both in favor of the intervention group (Table 3). No significant differences were noted in other measures (Table S2)**.**

For physiological measures, compared to the control group, intervention subjects had increased vitamin B_12_ levels after 18 months, increased hemoglobin, and reduced MCV at the 10-month period, which did not persist after 18 months, as well as a small decrease in HDL cholesterol, and a small increment of total cholesterol/ HDL ratio at the 10-month period post-intervention, which also did not persist after 18 months (Table 4). The intervention subjects also have significantly lower blood creatinine at the 10-month period compared to the control subjects. No significant differences were noted in other measures (Table S2).

### 5.4. Loss-to-Follow-up

**Figure 1:**
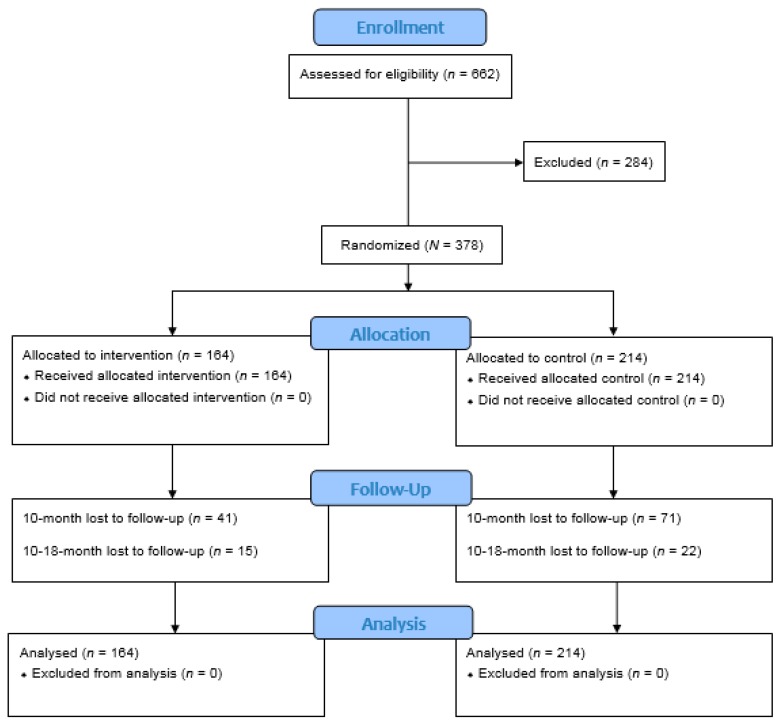
CONSORT 2010 Flow Diagram for SCOPE.

We reported two separate figures for participants who were loss-to-follow-ups (Figure 1 and Figure S1). The percentages of losses to follow-ups for individual measures was 32% and 39% for the composite measure. We performed non-responder analyses to examine if there were any baseline demographics that differentiated those retained in the intervention from participants who were loss-to-follow-up. At both subsequent time-points at 8 and 18-months, there were no significant differences in all the demographics for those retained versus loss-to-follow-up, except gender (more males were lost in the intervention arm, 10-month *p* = 0.005 and 18-month *p* = 0.011) and marital status (more non-married participants were lost in the control arm, 10-month *p* = 0.002 and 18-month *p* = 0.001) (Table S3).

## 6. Discussion

In this cluster RCT, a community-based intervention aimed at promoting self-care, SCOPE, had no effects on improving the measures of three common indicators of morbidities, such as blood pressure, diabetes, and COPD, either in a composite measure or as an individual measure. However, SCOPE improved three physiological and functional domains that are implicated in aging and geriatric syndromes, including: (1) increased lower body muscle strength (indicated by improved total time to complete the chair-stand test), (2) improved cardiopulmonary function (indicated by improved oxygen desaturation upon performing the chair-stand test), and (3) improved profile of blood biochemistry, represented by improved vitamin B_12_, hemoglobin concentration, mean corpuscular volume (MCV), and creatinine.

Though not focused primarily on specific health conditions of the participants, based on previous literature, improvement in understanding one’s role in one’s health may decrease the probability of experiencing trigger events that lead to dependency in old age [11] and admission to long-term care institutions [31], which we hypothesized could be achieved by a self-care interventional program like SCOPE. Taking into account the nuances in the nature of self-care versus CDSMP program, the differences in our findings with those of CDSMPs suggests that, to effect meaningful changes in disease-specific measures, general education about conditions and health behaviors, which were the cornerstones of SCOPE, is not sufficient.

On program implementation and fidelity, in SCOPE’s protocol, there was a linkage to the general practitioners after the participants were informed of having a particular condition, based on the tests. However, most of the participants did not follow-up with our referrals to visit the physicians. This issue has also been noted in previous studies. Even after receiving a clinical diagnosis, older adults are less likely to visit healthcare centers due to a variety of barriers [32,33]. Therefore, we postulate that, to improve morbidities-specific measures, an interventional program must first target patient activation, including the targeted and intensive aspects of CDSMP, and inclusion of primary care physicians in the program [34]. Implementation fidelity was cited as one of the most critical aspects of educational intervention [35].

The functional and physiological findings indicated that there were several notable and measurable benefits of SCOPE on preventing geriatric syndromes in older adults. Reduction in the total time to complete the chair-stand test indicates improved lower body muscle strength [36]. Lower body muscle strength decreases with age [37]. Hence, this improvement has implications in delaying the onset of disability and frailty, and, thereby, reduce the risk of older adults from falls [38] and maintain independence. Furthermore, lower body function is predictive of mortality [39]. Significantly less oxygen desaturation upon completing the chair-stand test also indicated that the participants in the treatment arm had higher exercise tolerance due to improved cardiopulmonary function [40]. Although non-significant upon performing Bonferroni correction, there was a trend of increased heart rate upon completing the chair-stand test in the SCOPE intervention group. Aging-induced changes in cardiovascular functions are partly due to a reduced maximal heart rate [41]. Conversely, enhanced cardiovascular functions have been shown to improve physical functions in older adults [42].

The vitamin B_12_ improvements in the intervention arm at the 18-month period is notable. Similar to our findings, improved nutritional biomarkers were reported previously after a nutrition education program [43]. After attending the intervention with nutrition classes, participants may have made better food choices, by increasing their intake of various macronutrients and micronutrients. Older adults generally have a lower B_12_ level due to atrophic gastritis impacting the absorption of B_12_ [44]. Thus, an improved B_12_ level has important clinical implications for older adults, including reduced risks of cognitive impairment, sarcopenia, dynapenia, and fracture.

Increased hemoglobin concentration and decreased MCV may reflect the short-term effect of SCOPE among the intervention subjects, since this difference did not persist throughout the 18-month period. Additionally, vitamin B_12_ deficiency was associated with a significant decrease in hemoglobin concentration [45]. Furthermore, the rise in hemoglobin and a decrease in MCV also corresponded well with the finding of increased B_12_ levels shown in this study, as these markers are linked in the same molecular pathway. Some of the plausible mechanisms for these improvements could be due to the specific contents in SCOPE, such as nutrition and exercise classes. It is noteworthy that the nature of the secondary measures in SCOPE was exploratory and hypothesis-generating. Hence, we note that, without explicitly examining the use of supplements and changes in lifestyles, the mechanisms leading to improvements in the secondary outcomes warrant future investigations. Having said that, we have shown insignificant changes in medication intake for each of the three chronic conditions and the total medication intake across the intervention period (Table S4). Furthermore, we observed no significant changes in smoking status across time-points, while physical activities and social activities were significantly lower (p>0.001) and higher (*p* = 0.02) in the intervention group, respectively (Table S4). Despite these findings, the multivariate results were unaffected by including these variables as covariates. Other physiological and functional measures did not improve following the intervention. Again, as with the lack of effect on condition-specific measures, one plausible interpretation for the paucity of measurable changes is that SCOPE did not involve the level of direct intervention required to improve the measures.

This study has several limitations. With regard to the B_12_ findings, we did not examine other laboratory results related to B_12_ metabolisms, such as folate, homocysteine, and methylmalonic acid. This metabolic pathway is worth further investigations since atrophic gastritis may be sufficiently common to justify preemptive use of oral B_12_ supplementation in this population. Additionally, the attrition rate of 39% was higher than anticipated (Figure 1), and approximately doubled the expected total attrition rate of 20%. This unexpected attrition rate might have rendered the primary measures under-powered on top of the issue of heterogeneity in participant characteristics. Nonetheless, retention has been widely acknowledged as a challenging issue in any RCT, especially when the program involves community-based large-scale implementation involving multiple ethnic groups. Nonetheless, the high attrition rate of 39% was comparable to and, in some cases, lower than previous studies with low-income sample populations. For example, the attrition rate of SCOPE (39%) was lower than those of studies conducted with low-income African Americans (61%) [46] and low-income adult males with a high proportion of African Americans (45.45%) [47]. In addition, participants’ heterogeneity could have masked the effects of SCOPE. We also acknowledge that statistically significant secondary measures might represent chance findings due to the issue of multiple testing. However, it has been noted that Bonferroni’s correction is conservative and inflates the type II error rate, and, hence, avoids spurious significant results [28]. Furthermore, we argue that these statistically significant measures were more sensitive to the effects of the intervention as compared to other measures [48]. Lastly, we did not examine a number of potential confounders, which could have contributed to the improvements in the measures observed, including intake of supplements and other treatments, such as physical therapy and traditional Chinese medicine. Although encouraging, the positive findings shown in this study need to be confirmed in future studies.

There are several strengths to this study. One of the main contributions of SCOPE is elucidating the effects elicited by a self-care program. By performing a study relying on self-care rather than self-management, we could compare and contrast findings gleaned from these two closely related yet distinct behavioral interventions. Second, SCOPE had a study design of a large-scaled cluster RCT that measured and, thus, minimized potential unaccounted variables and bias while remaining practical and generalizable. Third, the inclusion of the 18-month follow-up period allowed us to examine the long-term effects of the intervention. This is in contrast with a single follow-up within a shorter period, as performed by most of the CDSMPs. Fourth, SCOPE was also conducted in low income, multi-ethnic, and culturally diverse populations. Previous studies, including CDSMPs, rarely address the applicability of the programs to participants of different socioeconomic status (SES), cultural, and ethical backgrounds. Lastly, by including a wide range of physiological and functional measures, we showed pilot data on the plausible effects of self-care and expanded objective outcome measures for future examinations of self-care programs.

## 7. Conclusions and Implications

In all, despite the non-significant difference in the primary outcome, we showed evidence on the positive and encouraging effects of SCOPE on a number of health dimensions, represented by multiple functional and physiological measures that reflect multiple systems implicated in geriatric syndromes. The improved measures included lower body muscle strength, improved cardiopulmonary functions, and improved multiple indicators of the blood biochemistry profile. Further investigations of self-care programs in the community are needed to replicate and validate these objective findings. Taken together with the finding of a primary outcome measure, we further propose that combining a self-care program with a disease-specific and a disease-focused CDSMP program could potentially confer optimal benefits to community-dwelling older adults with morbidities. A crucial future consideration for either programs would be to involve general practitioners in the community, and, thus, introduce a community-based program that is not hospital-bound.

## Figures and Tables

**Table 1:** Demographic characteristics of participants in the intervention and control arms at baseline.

Demographics Characteristics	Intervention (*n* = 164) mean ± SD *n* (%)	Control (*n* = 214) mean ± SD *n* (%)	*p*-Value
Age (in years)	72.22 ± 7.79	72.16 ± 8.24	0.942
Age group (in years)			
55–60	12 (7.3)	15 (7)	0.611
61–65	23 (14.0)	37 (17.3)	
66–70	31 (18.9)	39 (18.3)	
71–75	42 (25.6)	47 (22.1)	
76–80	35 (21.3)	35 (16.4)	
81–85	14 (8.5)	28 (13.1)	
≥85	7 (4.3)	12 (5.6)	
Gender			
Female	104 (63.3)	147 (68.7)	0.282
Male	60 (36.6)	67 (31.3)	
Ethnicity			0.032 *
Chinese	144 (87.8)	170 (79.4)	
Non-Chinese	20 (12.2)	44 (20.6)	
Education			0.260
No formal education and primary school education only	30 (18.3)	30 (14)	
Secondary school education and above	134 (81.7)	184 (86)	
Marital Status			<0.001 ***
Married	72 (43.9)	137 (64)	
Not married	92 (56.1)	77 (36)	
Working Status			0.400
Holding full-time or part-time job	18 (11)	18 (8.4)	
Not working/retiree/housewife	146 (89)	196 (91.6)	
Housing Type			0.802
1–3 room public housing	149 (90.9)	196 (91.6)	
≥4 room public housing /private housing	15 (9.1)	18 (8.4)	
BMI (kg/m^2^)	24.13 ± 4.73	24.52 ± 4.98	0.447
Number of morbidities	2.46 ± 1.58	2.31 ± 1.62	0.386

Footnotes: BMI = body mass index. * *p* ≤ 0.05, *** *p* ≤ 0.001. Bold values indicate significant *p*-value upon performing Bonferroni correction.

**Table 2:** Adjusted models for primary outcome with two sensitivity analyses performed.

Composite Measures	Time-Points	Intervention Group	Control Group	Estimate (SE)	95% CI of the Estimates	*t*	*p*−Value
		Cases with at Least One Improvement/Total Number of Cases (% of Cases with at Least One Improvement)	Cases with at Least One Improvement/Total Number of Cases (% of Cases with at Least One Improvement)				
Original dataset	10-month	36/123 (29.3%)	40/143 (28%)	−0.067	−0.577 to 0.442	−2.06	0.795
	18-month	30/108 (27.8%)	29/121 (24%)	−0.214	−0.782 to 0.354	−0.74	0.459
Missing values replaced with 1	10-month	77/164 (46%)	111/214 (51.9%)	0.191	−0.341 to 0.7237	0.75	0.463
	18-month	86/164 (52.4%)	122/214 (57%)	0.179	−0.354 to 0.712	0.70	0.493
Missing values replaced with 0	10-month	36/164 (22%)	40/214 (18.7%)	−0.255	−1.027 to 0.518	−0.70	0.495
	18-month	30/164 (18.3%)	29/214 (13.6%)	−0.413	−1.215 to 0.389	−1.07	0.296

Footnote: CI = Confidence Interval.

**Table 3:** Adjusted models for secondary outcomes: functional measures.

Measures	Time-Points	Intervention	95% CI	Control	95% CI	Estimate	95% CI of the Estimates	*t*	*p*-Value
(Units)		Mean ± SE (*N*)		Mean ± SE (*N*)		(SE)			
Time to completing chair-stand test (seconds)	Baseline	12.37 (0.34)	11.70 to 13.04	11.93 (0.32)	11.30 to 12.57	Reference	Reference	Reference	Reference
	10-month	10.27 (0.36)	9.57 to 10.98	12.29 (0.35)	11.61 to 12.98	2.45 (0.41)	1.65 to 3.25	5.99	**<0.001**
	18-month	10.04 (0.37)	9.21 to 10.77	12.16 (0.36)	11.45 to 12.87	2.55 (0.46)	1.64 to 3.45	5.53	**<0.001**
Oxygen desaturation after chair-stand test (%)	Baseline	−0.23 (0.15)	−0.52 to 0.06	0.08 (0.14)	−0.19 to 0.35	Reference	Reference	Reference	Reference
	10-month	−0.06 (0.16)	−0.36 to 0.25	−0.41 (0.15)	−0.71 to −0.12	−0.66 (0.21)	−1.08 to −0.25	0.28	**0.002**
	18-month	−0.04 (0.16)	−0.36 to 0.28	−0.47 (0.16)	−0.78 to −0.17	−0.74 (0.22)	−1.17 to −0.32	0.24	**0.001**
Difference in heart rate after chair-stand test (BPM)	Baseline	10.80 (0.72)	9.38 to 12.22	10.79 (0.68)	9.45 to 12.14	Reference	Reference	Reference	Reference
	10-month	12.38 (0.77)	10.87 to 13.89	9.72 (0.75)	8.25 to 11.18	−2.66 (0.96)	−4.54 to −0.78	0.006	0.006
	18-month	10.31 (0.79)	8.75 to 11.86	10.91 (0.77)	9.39 to 12.42	0.60 (1.03)	−1.41 to 2.62	0.558	0.558

Footnotes: 95% CI = 95% confidence interval. SE = Standard error. Bold values indicate significant *p*-value upon performing Bonferroni correction (*p* < 0.005).

**Table 4:** Adjusted models for secondary outcomes: physiological measures.

Measures	Time-Points	Intervention	95% CI	Control	95% CI	Estimate	95% CI of the Estimates	*t*	*p*-Value
(Units)		Mean ± SE (*N*)		Mean ± SE (*N*)		(SE)			
Vitamin B12 (pmol/L)	Baseline	291.57 (12.52)	266.99 to 316.15	289.49 (11.97)	265.97 to 313.00	Reference	Reference	Reference	Reference
	10-month	336.46 (13.14)	310.66 to 362.26	321.99 (12.76)	296.93 to 347.05	−12.39 (13.53)	−38.96 to 14.19	−0.915	0.360
	18-month	397.22 (13.49)	370.74 to 423.71	329.31 (13.13)	3030.52 to 355.10	−65.83 (15.73)	−96.71 to −34.96	−4.185	**<0.001**
Hemoglobin concentration (g/dL)	Baseline	13.26 (0.07)	13.13 to 13.40	13.30 (0.07)	13.17 to 13.43	Reference	Reference	Reference	Reference
	10-month	13.50 (0.07)	13.40 to 13.64	13.26 (0.07)	13.13 to 13.40	−0.27 (0.07)	−0.41 to −0.12	−3.63	**<0.001**
	18-month	13.28 (0.07)	13.14 to 13.42	13.29 (0.07)	13.15 to 13.43	−0.03 (0.09)	−0.19 to 0.14	−0.19	0.758
MCV (fl)	Baseline	90.04 (0.25)	89.55 to 90.53	89.94 (0.24)	89.47 to 90.41	Reference	Reference	Reference	Reference
	10-month	89.86 (0.26)	89.35 to 90.38	90.69 (0.25)	90.19 to 91.19	0.93 (0.27)	0.39 to 1.47	3.39	**0.001**
	18-month	89.27 (0.27)	88.74 to 89.80	89.77 (0.26)	89.26 to 90.29	0.60 (0.32)	−0.20 to 1.23	1.90	0.058
HDL Cholesterol (mmol/L)	Baseline	1.52 (0.01)	1.50 to 1.55	1.52 (0.01)	1.50 to 1.54	Reference	Reference	Reference	Reference
	10-month	1.49 (0.01)	1.46 to 1.52	1.56 (0.01)	1.54 to 1.59	0.08 (0.02)	0.05 to 0.11	4.53	**<0.001**
	18-month	1.51 (0.01)	1.48 to 1.54	1.51 (0.01)	1.48 to 1.53	0.01 (0.02)	−0.03 to 0.04	0.04	0.969
Total Cholesterol/HDL Cholesterol (mmol/L)	Baseline	3.48 (0.04)	3.40 to 3.56	3.50 (0.04)	3.43 to 3.57	Reference	Reference	Reference	Reference
	10-month	3.54 (0.04)	3.46 to 3.62	3.40 (0.04)	3.32 to 3.48	−0.15 (0.05)	−0.25 to −0.06	−3.29	**0.001**
	18-month	3.46 (0.04)	3.38 to 3.54	3.46 (0.04)	3.38 to 3.54	−0.02 (0.05)	−0.41 to −0.69	0.69	0.08
Creatinine (umol/L)	Baseline	75.98 (0.96)	74.10 to 77.86	76.26 (0.91)	74.48 to 78.05	Reference	Reference	Reference	Reference
	10-month	80.95 (1.02)	78.95 to 82.95	85.15 (0.99)	83.22 to 87.09	3.92 (1.27)	1.42 to 6.43	3.08	**0.002**
	18-month	77.30 (1.04)	75.25 to 79.35	77.16 (1.01)	75.17 to 79.15	−0.42 (1.36)	−3.08 to 2.25	−0.31	0.756
Waist circumference (cm)	Baseline	94.34 (11.94)	70.89 to 117.79	95.94 (11.30)	73.75 to 118.13	Reference	Reference	Reference	Reference
	10-month	94.81 (12.78)	69.72 to 119.89	132.22 (12.35)	107.97 to 156.47	35.82 (17.16)	2.12 to 69.52	2.087	0.037
	18-month	117.55 (13.03)	91.98 to 143.12	123.21 (12.71)	98.27 to 148.15	4.06 (17.42)	−30.12 to 38.25	0.233	0.816

Footnotes: MCV: Mean corpuscular volume. HDL: high-density lipoprotein. 95% CI = 95% confidence interval. SE = Standard error. Bold values indicate significant *p*-value upon performing Bonferroni Correction (*p* < 0.005).

## Supplementary Materials

The following are available online at https://www.mdpi.com/2077-0383/9/3/885/s1, Figure S1: CONSORT 2010 Flow Diagram for SCOPE (individual measures); Table S1: Number of participants with each of the chronic condition and with at least one chronic conditions at baseline; Table S2: Unadjusted models for physiological and functional measures; Table S3: Non-responder analyses of the composite measure for participants retained versus loss to follow up. Table S4: Statistical models for changes in medication intakes and lifestyle measures across intervention time-points.
