# Supplementary material for: Effects of Self-Care for Older PErsons (SCOPE) on Functional and Physiological Measures: A Cluster Randomized Controlled Trial
Source: J Clin Med. 2020 Mar 24;9(3):885. doi: 10.3390/jcm9030885 (PMC7141527; doi:10.3390/jcm9030885)
Supplement: Supplementary file 1 [file jcm-09-00885-s001.pdf]

Figure S1. CONSORT 2010 Flow Diagram for SCOPE (individual measures).

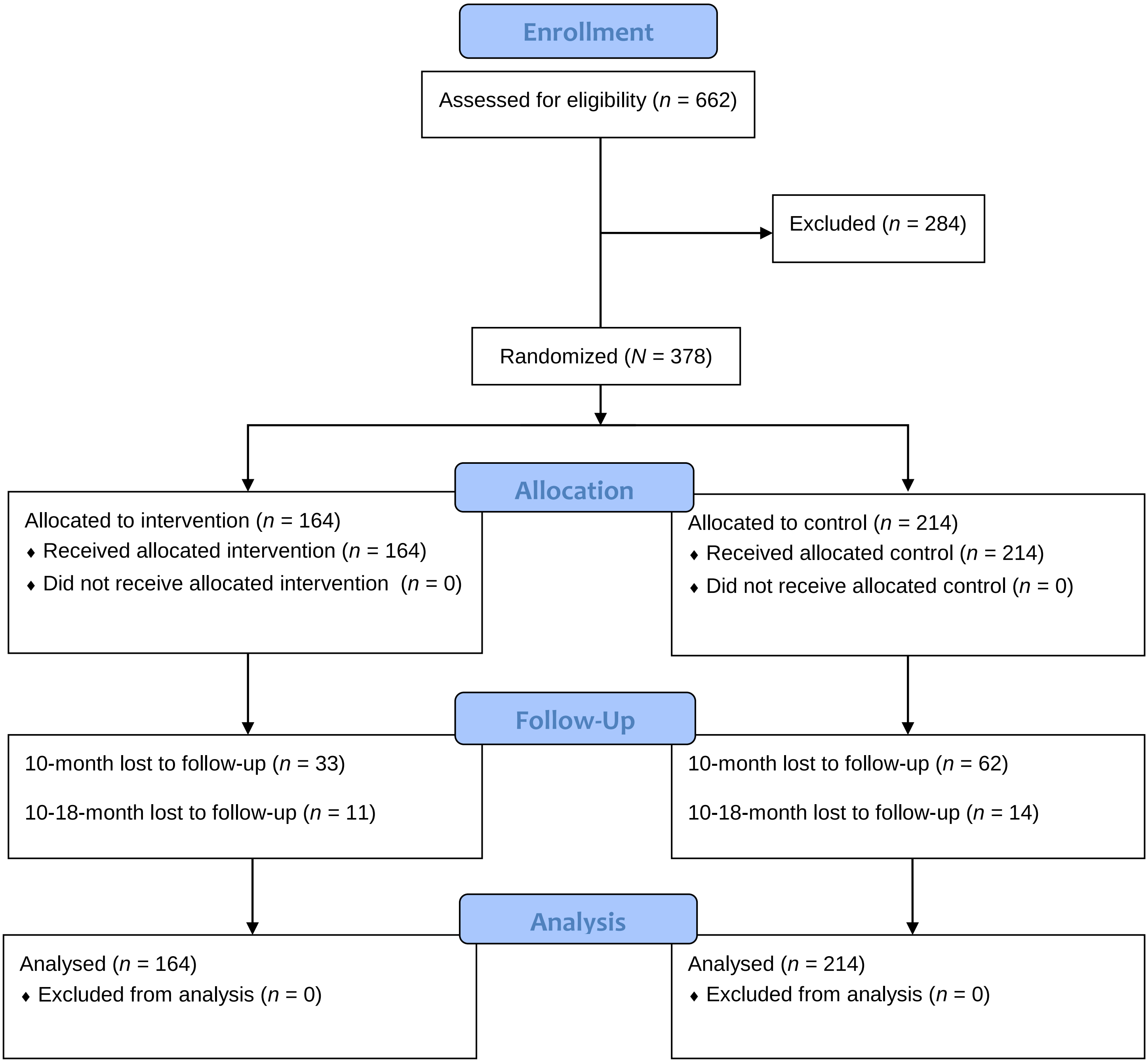

Table S1. Number of participants with each of the chronic condition and with at least one chronic conditions at baseline.

SCOPE Target Chronic Conditions	Identified Prior to or during the Study	Intervention Group (n = 164), %	Control Group (n = 214), %
Hypertension only	Prior to the study<br>During the study	99 (60.4)<br>26 (15.9)	123 (57.5)<br>46 (21.5)
Diabetes only	Prior to the study<br>During the study	38 (23.2)<br>17 (10.4)	49 (22.9)<br>19 (8.9)
COPD only	Prior to the study<br>During the study	12 (7.3)<br>110 (67.1)	10 (4.7)<br>132 (61.7)
Had at least one or more of hypertension, diabetes and COPD	Combined	157 (97.5)*	203 (96.7%)**

Footnotes: Participants identified prior to the study was by self-report of having the condition or by self-report of medication intake; participants identified during the study was by baseline biomarker measurements; combined = by both self-report and biomarker measurements * = 3 participants with readings missing, ** = 4 participants with readings missing.

Table S2. Unadjusted models for physiological and functional measures.

Laboratory Measures (units)	Time-Points	Intervention Mean ±SE (N)	Control Mean ± SE (N)	*p*-Value
HbA1C (%)	Baseline<br>10-month<br>18-month	6.48 ±1.29 (N=163)<br>6.40 ±1.21 (N=131)<br>6.42 ±1.21 (N=120)	6.46 ±1.20 (N=214)<br>6.35 ±1.20 (N=152)<br>6.40 ±1.20 (N=138)	Reference<br>0.734<br>0.948
Vitamin B12 (pmol/L)	Baseline<br>10-month<br>18-month	301.03 ±205.46 (N=164)<br>351.63 ±211.91 (N=132)<br>410.03 ±223.84 (N=120)	306.81 ±158.24 (N=214)<br>340.49 ±182.03 (N=152)<br>345.22 ±136.96 (N=138)	Reference<br>0.605<br>**0.001****
Hemoglobin concentration (g/dL)	Baseline<br>10-month<br>18-month	13.11 ±1.51 (N=163)<br>13.31 ±1.40 (N=132)<br>13.12 ±1.39 (N=120)	13.30 ±1.30 (N=214)<br>13.41 ±1.30 (N=152)<br>13.42 ±1.32 (N=138)	Reference<br>**0.002****<br>0.846
MCV (fl)	Baseline<br>10-month<br>18-month	90.65 ±5.78 (N=163)<br>90.74 ±5.38 (N=132)<br>90.32 ±5.18 (N=120)	89.67 ±6.27 (N=214)<br>89.99 ±6.59 (N=152)<br>89.17 ±6.42 (N=138)	Reference<br>**0.005****<br>0.280
Platelets (x10^9/L)	Baseline<br>10-month<br>18-month	250.14 ±59.45 (N=214)<br>252.98 ±60.34 (N=132)<br>264.60 ±64.00 (N=120)	254.37 ±67.88 (N=214)<br>260.63 ±68.43 (N=152)<br>268.54 ±82.53 (N=138)	Reference<br>0.298<br>0.945
WBC count (x10^9/L)	Baseline<br>10-month<br>18-month	6.68 ±1.76 (N=163)<br>6.80 ±1.62 (N=132)<br>6.79 ±1.63 (N=120)	6.65 ±1.66 (N=214)<br>6.79 ±1.72 (N=152)<br>6.80 ±1.61 (N=138)	Reference<br>0.455<br>0.920
Total Cholesterol (mmol/L)	Baseline<br>10-month<br>18-month	4.96 ±1.02 (N=164)<br>4.91 ±1.06 (N=132)<br>4.88 ±0.97 (N=120)	5.07 ±1.02 (N=214)<br>5.05 ±1.09 (N=152)<br>5.01 ±1.03 (N=138)	Reference<br>0.506<br>0.832
HDL Cholesterol (mmol/L)	Baseline<br>10-month<br>18-month	1.54 ±0.43 (N=164)<br>1.51 ±0.41 (N=132)<br>1.53 ±0.44 (N=120)	1.48 ±0.35 (N=214)<br>1.53 ±0.40 (N=152)<br>1.47 ±0.35 (N=138)	Reference<br>**<0.001*****<br>0.857
LDL Cholesterol (mmol/L)	Baseline<br>10-month	2.79 ±0.84 (N=163)<br>2.79 ±0.87 (N=131)	2.95 ±0.93 (N=213)<br>2.87 ±0.93 (N=151)	Reference<br>0.456

	18-month	2.73±0.77 (N=119)	2.91±0.89 (N=138)	0.758
Total Cholesterol/ HDL Cholesterol (mmol/L)	Baseline<br>10-month<br>18-month	3.41±0.96 (N=164)<br>3.43±1.08 (N=132)<br>3.36±0.98 (N=120)	3.59±1.00 (N=214)<br>4.43±0.87 (N=152)<br>3.53±0.91 (N=138)	Reference<br>**0.003****<br>0.967
Triglycerides (mmol/L)	Baseline<br>10-month<br>18-month	1.38±0.70 (N=164)<br>1.37±0.83 (N=132)<br>1.37±0.85 (N=120)	1.44±0.78 (N=214)<br>1.44±0.72 (N=152)<br>1.38±0.57 (N=138)	Reference<br>0.547<br>0.591
Albumin (g/L)	Baseline<br>10-month<br>18-month	42.60±2.59 (N=164)<br>43.65±2.43 (N=132)<br>41.94±2.38 (N=120)	43.44±2.34 (N=214)<br>44.31±2.55 (N=152)<br>42.76±2.55 (N=138)	Reference<br>0.755<br>0.936
Creatinine (umol/L)	Baseline<br>10-month<br>18-month	79.79±46.81 (N=164)<br>87.00±75.56 (N=132)<br>80.26±55.26(N=120)	75.43±30.20 (N=214)<br>83.77±38.18 (N=152)<br>72.70±26.29 (N=138)	Reference<br>**0.035***<br>0.838
Urine microalbumin to creatinine ratio (ACR) (mg/dL)	Baseline<br>10-month<br>18-month	0.84±0.23 (N=164)<br>0.99±2.45 (N=132)<br>1.15±2.21 (N=122)	0.84±0.28 (N=214)<br>0.76±1.84 (N=152)<br>0.87±2.24 (N=137)	Reference<br>0.279<br>0.235
Blood pressure -Systolic (mmHg)	Baseline<br>10-month<br>18-month	140.18±02.24 (N=164)<br>143.78±21.18 (N=132)<br>139.98±19.25 (N=122)	141.85±19.44 (N=214)<br>140.53±18.08 (N=152)<br>136.61±17.79 (N=137)	Reference<br>0.130<br>0.081
Blood pressure -Diastolic (mmHg)	Baseline<br>10-month<br>18-month	77.88±12.38 (N=164)<br>78.68±10.46 (N=132)<br>77.52± 10.93 (N=122)	78.14±11.97 (N=214)<br>78.12±9.57 (N=152)<br>76.30±10.21 (N=137)	Reference<br>0.824<br>0.292
Pulse (BPM)	Baseline<br>10-month<br>18-month	70.61±11.83 (N=164)<br>70.48±10.46 (N=132)<br>71.58±11.58 (N=122)	71.06±11.92 (N=210)<br>71.21±10.99 (N=151)<br>71.61±11.78 (N=136)	Reference<br>0.929<br>0.371
Peak expiratory flow reading (L/min)	Baseline<br>10-month<br>18-month	230.25±95.13 (N=163)<br>244.18±96.43 (N=130)<br>243.43±104.52 (N=118)	225.25±92.04 (N=214)<br>263.08±101.46 (N=152)<br>255.61±122.66 (N=134)	Reference<br>0.303<br>0.439
BMI (kg/m2)	Baseline<br>10-month	24.13± 4.73 (N=163)<br>24.56±4.66 (N=128)	24.52± 4.98 (N=210)<br>24.55±4.87 (N=143)	Reference<br>0.312

	18-month	24.34±4.52 (N=117)	25.38±13.49 (N=133)	0.392
Weight circumference (cm)	Baseline<br>10-month<br>18-month	88.28±12.68 (N=164)<br>95.38±80.30 (N=131)<br>118.50±163.22 (N=122)	87.88±12.44 (N=214)<br>124.73±178.19 (N=152)<br>128.28±187.37 (N=137)	Reference<br>**0.023***<br>0.880
Time for completing chair-stand test (seconds)	Baseline<br>10-month<br>18-month	13.39±4.55 (N=150)<br>10.86±2.23 (N=121)<br>10.66±3.61 (N=110)	11.78±3.90 (N=194)<br>11.79±3.94 (N=144)<br>11.56±4.53 (N=128)	Reference<br>**<0.001*****<br>**<0.001*****
Difference in oxygen saturation after chair-stand test (%)	Baseline<br>10-month<br>18-month	-0.46±1.62 (N=150)<br>-0.26±1.59 (N=121)<br>-0.26±1.59 (N=110)	0.10±1.33 (N=195)<br>-0.33±1.34 (N=144)<br>-0.41±1.27 (N=128)	Reference<br>0.068<br>**0.007****
Difference in heart rate after chair-stand test (BPM)	Baseline<br>10-month<br>18-month	9.93±7.99 (N=150)<br>11.64±6.92 (N=121)<br>9.61±6.87 (N=110)	10.51±6.32 (N=195)<br>9.50±6.74 (N=144)<br>10.71±8.03 (N=128)	Reference<br>0.054<br>0.532
Hand grip strength (kg)	Baseline<br>10-month<br>18-month	20.98±6.79 (N=155)<br>22.66±7.33 (N=126)<br>21.69±5.83 (N=111)	19.77±6.32 (N=203)<br>22.34±7.59 (N=150)<br>21.38±7.26 (N=142)	Reference<br>0.290<br>0.122
iADL	Baseline<br>10-month<br>18-month	0.330±0.08 (N=164)<br>0.28±0.08 (N=137)<br>0.47±0.09 (N=130)	0.33±0.07 (N=214)<br>0.30±0.07 (N=185)<br>0.32±0.08 (N=162)	Reference<br>0.852<br>0.304
SRH	Baseline<br>10-month<br>18-month	78.72±27.98 (N=164)<br>73.16±29.53 (N=137)<br>72.34±30.64 (N=130)	77.39±24.50 (N=214)<br>65.27±25.57 (N=214)<br>14.20±27.04 (N=214)	Reference<br>0.845<br>0.194

Footnotes: HbA1C: hemoglobin A1c; MCV: Mean corpuscular volume; HDL: high-density lipoprotein, LDL: low-density lipoprotein, RBC: red blood cell, WBC: white blood cell, ACR: albumin to creatinine ratio; BMI: body-mass index; BPM= beats per minute; iADL: independent activities of daily living; SRH: self-rated health; * $p \leq 0.05$,** $p \leq 0.01$,*** $p \leq 0.001$.

Table S3. Non-responder analyses of the composite measure for participants retained versus loss to follow up.

a. Gender difference, 10-month

Control or Intervention				Participants Retained or Loss to Follow-Up		Total	*p*-Value
				Loss to follow-up	Retained		
Control	Gender	Male	Count	19	48	67	0.350
			% within Gender	28.4%	71.6%	100.0%	
			% within Participants retained or loss to follow-up	26.8%	33.6%	31.3%	
			% of Total	8.9%	22.4%	31.3%	
		Female	Count	52	95	147	
			% within Gender	35.4%	64.6%	100.0%	
			% within Participants retained or loss to follow-up	73.2%	66.4%	68.7%	
			% of Total	24.3%	44.4%	68.7%	
	Total		Count	71	143	214	
			% within Gender	33.2%	66.8%	100.0%	
			% within Participants retained or loss to follow-up	100.0%	100.0%	100.0%	
			% of Total	33.2%	66.8%	100.0%	
Intervention	Gender	Male	Count	23	37	60	****0.005**
			% within Gender	38.3%	61.7%	100.0%	
			% within Participants retained or loss to follow-up	56.1%	30.1%	36.6%	
			% of Total	14.0%	22.6%	36.6%	
		Female	Count	18	86	104	
			% within Gender	17.3%	82.7%	100.0%	
			% within Participants retained or loss to follow-up	43.9%	69.9%	63.4%	
			% of Total	11.0%	52.4%	63.4%	
	Total		Count	41	123	164	
			% within Gender	25.0%	75.0%	100.0%	
			% within Participants retained or loss to follow-up	100.0%	100.0%	100.0%	
			% of Total	25.0%	75.0%	100.0%	

b. Gender difference, 18-month

Control or Intervention				Participants Retained or Loss to Follow-Up		Total	*p*-Value
				Loss to Follow-Up	Retained		
Control	Gender	Male	Count	29	38	67	1.000
			% within Gender	43.3%	56.7%	100.0%	
			% within Participants retained or loss to follow-up	31.2%	31.4%	31.3%	
			% of Total	13.6%	17.8%	31.3%	
		Female	Count	64	83	147	
			% within Gender	43.5%	56.5%	100.0%	
			% within Participants retained or loss to follow-up	68.8%	68.6%	68.7%	
			% of Total	29.9%	38.8%	68.7%	
	Total		Count	93	121	214	
			% within Gender	43.5%	56.5%	100.0%	
			% within Participants retained or loss to follow-up	100.0%	100.0%	100.0%	
			% of Total	43.5%	56.5%	100.0%	
Intervention	Gender	Male	Count	28	32	60	***0.011**
			% within Gender	46.7%	53.3%	100.0%	
			% within Participants retained or loss to follow-up	50.0%	29.6%	36.6%	
			% of Total	17.1%	19.5%	36.6%	
		Female	Count	28	76	104	
			% within Gender	26.9%	73.1%	100.0%	
			% within Participants retained or loss to follow-up	50.0%	70.4%	63.4%	
			% of Total	17.1%	46.3%	63.4%	
	Total		Count	56	108	164	
			% within Gender	34.1%	65.9%	100.0%	
			% within Participants retained or loss to follow-up	100.0%	100.0%	100.0%	
			% of Total	34.1%	65.9%	100.0%	

c. Marital Status difference, 10-month

Control or Intervention				Participants Retained Or Loss To Follow-Up		Total	
				Loss to Follow-Up	Retained		
Control	Marital Status	Unmarried	Count	36	41	77	****0.002**
			% within Marital Status: Married	46.8%	53.2%	100.0%	
			% within Participants retained or loss to follow-up	50.7%	28.7%	36.0%	
			% of Total	16.8%	19.2%	36.0%	
		Married	Count	35	102	137	
			% within Marital Status: Married	25.5%	74.5%	100.0%	
			% within Participants retained or loss to follow-up	49.3%	71.3%	64.0%	
			% of Total	16.4%	47.7%	64.0%	
	Total		Count	71	143	214	
			% within Marital Status: Married	33.2%	66.8%	100.0%	
			% within Participants retained or loss to follow-up	100.0%	100.0%	100.0%	
			% of Total	33.2%	66.8%	100.0%	
Intervention	Marital Status	Unmarried	Count	28	64	92	0.101
			% within Marital Status: Married	30.4%	69.6%	100.0%	
			% within Participants retained or loss to follow-up	68.3%	52.0%	56.1%	
			% of Total	17.1%	39.0%	56.1%	
		Married	Count	13	59	72	
			% within Marital Status: Married	18.1%	81.9%	100.0%	
			% within Participants retained or loss to follow-up	31.7%	48.0%	43.9%	
			% of Total	7.9%	36.0%	43.9%	
	Total		Count	41	123	164	

			% within Marital Status: Married	25.0%	75.0%	100.0%
			% within Participants retained or loss to follow-up	100.0%	100.0%	100.0%
			% of Total	25.0%	75.0%	100.0%

d. Marital Status difference, 18-month

**Control or Intervention**				**Participants Retained or Loss to Follow-Up**		**Total**	
				**Loss to Follow-Up**	**Retained**		
Control	Marital Status	Unmarried	Count	45	32	77	**0.001
			% within Marital Status: Married	58.4%	41.6%	100.0%	
			% within Participants retained or loss to follow-up	48.4%	26.4%	36.0%	
			% of Total	21.0%	15.0%	36.0%	
		Married	Count	48	89	137	
			% within Marital Status: Married	35.0%	65.0%	100.0%	
			% within Participants retained or loss to follow-up	51.6%	73.6%	64.0%	
			% of Total	22.4%	41.6%	64.0%	
	Total		Count	93	121	214	
			% within Marital Status: Married	43.5%	56.5%	100.0%	
			% within Participants retained or loss to follow-up	100.0%	100.0%	100.0%	
			% of Total	43.5%	56.5%	100.0%	
Intervention	Marital Status	Unmarried	Count	36	56	92	0.139
			% within Marital Status: Married	39.1%	60.9%	100.0%	
			% within Participants retained or loss to follow-up	64.3%	51.9%	56.1%	
			% of Total	22.0%	34.1%	56.1%	
		Married	Count	20	52	72	

	% within Marital Status: Married	27.8%	72.2%	100.0%
	% within Participants retained or loss to follow-up	35.7%	48.1%	43.9%
	% of Total	12.2%	31.7%	43.9%
Total	Count	56	108	164
	% within Marital Status: Married	34.1%	65.9%	100.0%
	% within Participants retained or loss to follow-up	100.0%	100.0%	100.0%
	% of Total	34.1%	65.9%	100.0%

Footnotes: *** denotes $p < 0.001$, ** denotes $p < 0.01$, * denotes $p < 0.05$.

Table S4. Statistical models for changes in medication intakes and lifestyle measures across intervention time-points.

**Measures**	**Time-points**	**Intervention (*N* = 164) Mean ±SE or *n* (%)**	**95% CI**	**Control (*N* = 214) Mean ± SE or *n* (%)**	**95% CI**	**Estimate (SE)**	**95% CI**	***t***	***p*-Value**
Total number of medications taken	Baseline<br>10-month<br>18-month	1.84 (0.12)<br>1.71 (0.12)<br>1.89 (0.12)	1.594 to 2.077<br>1.466 to1.949<br>1.637 to 2.120	1.83 (0.11)<br>1.68 (0.11)<br>1.80 (0.11)	1.616-2.038<br>1.471-1.894<br>1.592-2.015	Reference<br>-0.02<br>-0.07	Reference<br>-0.32 to 0.28<br>-0.36 to 0.24	Reference<br>-0.11<br>-0.43	Reference<br>0.28<br>0.24
Intake of medication for hypertension	Baseline<br>10-month<br>18-month	97 (59.1)<br>83 (50.6)<br>86 (52.4)	NA	123 (57.5)<br>109 (50.9)<br>101 (47.2)	NA	Reference<br>-0.08<br>0.14	NA	NA	Reference<br>0.78<br>0.63
Intake of medication for diabetes	Baseline<br>10-month<br>18-month	38 (23.2)<br>32 (19.5)<br>8 (4.9)	NA	48 (22.4)<br>43 (20.1)<br>6 (2.8)	NA	Reference<br>-0.08<br>0.15	NA	NA	Reference<br>0.83<br>0.68
Intake of medication for COPD & other chronic respiratory conditions	Baseline<br>10-month<br>18-month	10 (6.1)<br>7 (4.3)<br>0 (0)	NA	9 (4.2)<br>9 (4.2)<br>0 (0)	NA	Reference<br>-0.38<br>0.18	NA	NA	Reference<br>0.59<br>0.80
Smoking status	Baseline<br>10-month<br>18-month	19 (11.6)<br>13 (7.9)<br>15 (9.1)	NA	16 (7.5)<br>12 (5.6)<br>12 (5.6)	NA	Reference<br>-0.07<br>0.01	NA	NA	Reference<br>0.898<br>0.992
Physical activities	Baseline	4.22 (0.21)	3.80 to	2.79 (0.19)	2.43 to	Reference	Reference	Reference	Reference

	10-month<br>18-month	4.42 (0.23)<br>3.89 (0.24)	4.64<br>3.96 to<br>4.87<br>3.40 to<br>4.33	3.54 (0.20)<br>3.91 (0.21)	3.16<br>3.15 to<br>3.93<br>3.49 to<br>4.32	0.55<br>1.47	-0.09 to<br>1.19<br>0.80 to<br>2.13	1.68<br>4.32	0.09<br>**<0.001*****
Social activities	Baseline<br>10-month<br>18-month	5.90 (0.17)<br>6.25 (0.19)<br>7.58 (0.19)	5.57 to<br>6.24<br>5.89 to<br>6.62<br>7.20 to<br>7.96	5.92 (0.15)<br>6.58 (0.16)<br>6.75 (0.17)	5.62 to<br>6.21<br>6.26 to<br>6.90<br>6.41 to<br>7.09	Reference<br>0.32<br>-0.84	Reference<br>-0.37 to<br>1.00<br>-1.55 to -<br>0.14	Reference<br>0.91<br>-2.36	Reference<br>0.36<br>**0.02***

Footnotes: NA= not applicable; We measured the levels of physical activity by asking three questions: during the past week, even if it was not a typical week for you, how much total time did you spend on each of the following? 1) Stretching or strengthening exercises (Taiji, Qigong, range of motion, using weights, etc.), 2) Walk for exercise purposes, and 3) Participate in vigorous exercise (e.g. jogging, swimming, keep fit or dancing class, etc.). The participants rated the frequencies by which they participated in the three activities on a 5-point scale, ranging from 0 to 4, with a sum of 12. The higher score indicated higher levels of physical activity; We measured the levels of social activity by asking three questions: during the past week, how much total time did you spend on each of the following? 1) Stretching or strengthening exercises (Taiji, Qigong, range of motion, using weights, etc.), 2) Walk for exercise purposes, and 3) Participate in vigorous exercise (e.g. jogging, swimming, keep fit or dancing class, etc.). The participants rated the frequencies by which they participated in the three activities on a 5-point scale, ranging from 0 to 4, with a sum of 12. The higher score indicated higher levels of social activity; * $p \leq 0.05$,** $p \leq 0.01$,*** $p \leq 0.001$.
